# Use of Nonrecommended Drugs in Patients With Brugada Syndrome: A Danish Nationwide Cohort Study

**DOI:** 10.1161/JAHA.122.028424

**Published:** 2023-03-21

**Authors:** Camilla H. B. Jespersen, Johanna Krøll, Priya Bhardwaj, Carl Johann Hansen, Jesper Svane, Bo G. Winkel, Christian Jøns, Peter Karl Jacobsen, Jens Haarbo, Jens Cosedis Nielsen, Jens Brock Johansen, Berit T. Philbert, Sam Riahi, Christian Torp‐Pedersen, Lars Køber, Jacob Tfelt Hansen, Peter E. Weeke

**Affiliations:** ^1^ Department of Cardiology, The Heart Centre Copenhagen University Hospital, Rigshospitalet København Denmark; ^2^ Department of Cardiology Copenhagen University Hospital ‐ Herlev and Gentofte Hellerup Denmark; ^3^ Department of Cardiology Aarhus University Hospital Aarhus Denmark; ^4^ Department of Clinical Medicine Aarhus University Aarhus Denmark; ^5^ Department of Cardiology Odense University Hospital Odense Denmark; ^6^ Department of Cardiology Aalborg University Hospital Aalborg Denmark; ^7^ Department of Cardiology Nordsjaellands Hospital Hillerød Denmark; ^8^ Department of Public Health University of Copenhagen København Denmark; ^9^ Department of Forensic Medicine, Faculty of Medical Sciences University of Copenhagen København Denmark

**Keywords:** adverse drug events, BrS, pharmacotherapy, ventricular arrhythmia, Quality and Outcomes

## Abstract

**Background:**

Patients with Brugada syndrome (BrS) are recommended to avoid drugs that may increase their risk of arrhythmic events. We examined treatment with such drugs in patients with BrS after their diagnosis.

**Methods and Results:**

All Danish patients diagnosed with BrS (2006–2018) with >12 months of follow‐up were identified from nationwide registries. Nonrecommended BrS drugs were grouped into drugs to “avoid” or “preferably avoid” according to http://www.brugadadrugs.org. Cox proportional hazards analyses were performed to identify factors associated with any nonrecommended BrS drug use, and logistic regression analyses were performed to examine associated risk of appropriate implantable cardioverter defibrillator therapy, mortality, and a combined end point indicating an arrhythmic event of delayed implantable cardioverter defibrillator implantation, appropriate implantable cardioverter defibrillator therapy, and mortality. During a median follow‐up of 6.8 years, 93/270 (34.4%) patients with BrS (70.4% male, median age at diagnosis 46.1 years [interquartile range, 32.6–57.4]) were treated with ≥1 nonrecommended BrS drugs. No difference in any nonrecommended BrS drug use was identified comparing time before BrS diagnosis (12.6%) with each of the 5 years following BrS diagnosis (*P*>0.05). Factors associated with any nonrecommended BrS drug use after diagnosis were female sex (hazard ratio [HR]) 1.83 [95% CI, 1.15–2.90]), psychiatric disease (HR, 3.63 [1.89–6.99]), and prior use of any nonrecommended BrS drug (HR, 4.76 [2.45–9.25]). No significant association between any nonrecommended BrS drug use and implantable cardioverter defibrillator therapy (n=20/97, odds ratio [OR], 0.7 [0.2–2.4]), mortality (n=10/270, OR, 3.4 [0.7–19.6]), or the combined end point (n=38/270, OR, 1.7 [0.8–3.7]) was identified.

**Conclusions:**

One in 3 patients with BrS were treated with a nonrecommended BrS drug after BrS diagnosis, and a BrS diagnosis did not change prescription patterns. More awareness of nonrecommended drug use among patients with BrS is needed.

**Nonstandard Abbreviations and Acronyms**
BrS: Brugada syndrome

**Clinical Perspective**

**What Is New?**

- We found no difference in prescription patterns of nonrecommended drugs before and after a diagnosis of Brugada syndrome even though it is a Class I recommendation to avoid such drugs.
- Female sex, nonrecommended drug use before Brugada syndrome diagnosis, and psychiatric disease were significantly associated with nonrecommended drug use.

**What Are the Clinical Implications?**

- Patients should avoid intake of nonrecommended drugs so they do not further increase their risk of ventricular arrhythmias and sudden cardiac death.
- Physicians treating patients with Brugada syndrome should be aware of prescription patterns of nonrecommended drugs in these patients.

Brugada syndrome (BrS) is a rare inherited cardiac disease characterized by coved ST‐segment elevations in the right precordial leads in the ECG and a significant cause of sudden cardiac death in the young because of an increased risk of ventricular arrhythmias.^1^, ^2^ Symptoms of the disease are related to the development of malignant arrhythmias and can range from palpitations to syncope or sudden cardiac arrest. Treatment options for BrS are limited; current guidelines recommend implantation of an implantable cardioverter defibrillator (ICD) in patients with BrS and aborted cardiac arrest or documented sustained ventricular arrhythmias, and in case of recurrent ICD, shocks for ventricular fibrillation, quinidine, or catheter ablation.^3^ To lower the risk of ventricular arrhythmias, all patients with BrS are advised to avoid known modifiable risk factors of the disease, including intake of drugs that may increase the risk of ventricular arrhythmias (Class I recommendation).^3^ However, little is known about the overall use of nonrecommended BrS drugs among patients with BrS. To date, 1 small study by de Almeida Fernandes et al on 30 patients with BrS with ICDs examined self‐reported use of nonrecommended BrS drugs and found that approximately half of patients with BrS were in treatment with a drug that was not recommended.^4^ Collectively, very little information on the overall use of nonrecommended BrS drugs among patients with BrS exists, and it is not known if its use is associated with adverse outcomes.

To address these gaps in current knowledge, we performed a nationwide cohort study on all Danish patients diagnosed with BrS to determine use of nonrecommended BrS drugs in patients with BrS before and after their BrS diagnosis. Moreover, we evaluated potential factors associated with treatment with these drugs, and whether such treatment was associated with adverse events (ie, appropriate ICD therapy, mortality, or a combined end point indicating an arrhythmic event of delayed ICD implantation, appropriate ICD therapy, and mortality).

## METHODS

### Registries

All Danish citizens receive a unique and permanent identification number through the Civil Registration System upon birth or immigration. This allows for nationwide cross‐linkage among the Danish registries on an individual level. The Danish health care system is a government tax‐funded single payer system that guarantees unrestricted access to medical services.

Since 1978, all admissions to and discharges from Danish hospitals have been registered in the Danish National Patient Registry. For each admission and discharge, a primary diagnosis and relevant secondary diagnoses are registered according to the *International Classification of Diseases* (from 1994 and onwards: the *Tenth Revision* [*ICD‐10*]).^5^ The diagnosis code for BrS, DI472M, was introduced to this registry in 2006. Since 1995, all drug prescriptions from Danish pharmacies have been registered in the Danish National Prescription Register using the Anatomical Therapeutic Chemical system. Because of the partial reimbursement of drug expenses by the government‐financed health care system, it is a requirement by law for Danish pharmacies to register all dispensed prescriptions, making this register both accurate and valid.^6^ Since 1982, data on all pacemaker and ICD implantations performed in Denmark have been reported prospectively by the implanting physicians in the Danish Pacemaker and ICD Register.^7^ In addition to specific information on implantation, the register holds follow‐up information including appropriate and inappropriate device therapies.

For a subset of the patients followed at the specialized inherited cardiac disease clinic at Copenhagen University Hospital, Rigshospitalet, we also had access to additional clinical information through manual chart review. This included, for example, information on symptoms of the disease, results of genetic testing, and date of diagnosis.

Efforts were made not to report data that may aid in the identification of individuals, because Danish law prohibits reporting of low group numbers from the nationwide registries (n≤3); thus, such low group numbers were replaced with “≤3” throughout the article. The exact numbers are known to the investigators. Because of restrictions related to Danish law and protecting patient privacy, the combined set of data used in this study can only be made available through a trusted third party, Statistics Denmark. Data will be shared on request to the corresponding author with permission from Statistics Denmark. More information regarding data access is available at https://www.dst.dk/en/TilSalg/Forskningsservice.

### Study Population

We identified all Danish patients with a first‐time *ICD‐10* code of DI472M between 2006 and 2018 through the Danish National Patient Registry, and they were followed until December 31, 2019, ensuring that all patients were eligible for at least 12 months follow‐up. In Denmark, all patients with BrS have their diagnosis verified by cardiologists specializing in inherited cardiac diseases, and they are followed at clinics specializing in inherited cardiac diseases. Patients are diagnosed with BrS according to current guidelines.^3^ In brief, the guidelines include the following: in the presence of ST‐segment elevation with type 1 morphology ≥2 mm in 1 or more leads among the right precordial leads V1 and/or V2 positioned in the second, third, or fourth intercostal space; occurring either spontaneously, in relation to fever, or after provocative drug testing with intravenous administration of sodium channel blockers (such as ajmaline or flecainide). If the ST‐segment elevations do not occur spontaneously, other clinical features such as documented ventricular arrhythmia, arrhythmic syncope, or relevant family history are required to make the diagnosis. Brugada phenocopies (ie, other causes of ST‐segment elevation in the right precordial leads) must be excluded before diagnosis.

#### Validation of the BrS Diagnosis Code in Danish Registries

In relation to the present study, we manually reviewed the charts of patients assigned a BrS diagnosis at Copenhagen University Hospital, Rigshospitalet, to ensure the validity of the *ICD‐10* code specifically allocated to define BrS (ie, DI472M). We reviewed 72 patients with BrS, and the positive predictive value was 95.8%.

### BrS Disease Manifestation

We determined BrS disease manifestation according to information from the medical records (where available), through hospital discharge diagnoses from the Danish National Patient Registry for cardiac events in relation to time of BrS diagnosis, or through indication for ICD implantation (before diagnosis) registered in the Danish Pacemaker and ICD Register. We categorized patients with BrS according to their disease manifestation at the time of diagnosis as “symptomatic” (ie, had experienced aborted cardiac arrest, ventricular tachycardia, or syncope) or as “asymptomatic or unspecified disease manifestation.” Asymptomatic patients were defined as patients having neither any clinical symptoms registered through chart review nor any outpatient clinic or in‐hospital diagnoses for cardiac events before the time of diagnosis. Thus, only patients eligible for full chart review were qualified to be truly asymptomatic, as done previously.^8^, ^9^, ^10^ Patients with ventricular tachycardia or syncope and patients with aborted cardiac arrest were defined through chart review, hospital discharge codes (see Table S1 for specific *ICD‐10* codes), and/or indication for ICD implantation. Patients with inconclusive disease manifestation according to registries and where chart review was not possible were categorized as unspecified.

### Nonrecommended BrS Drugs

Drugs that should be avoided because of possibly inducing ST‐segment elevation in the right precordial leads and ventricular arrhythmias were identified according to http://www.brugadadrugs.org (accessed August 1, 2022) using relevant Anatomical Therapeutic Chemical codes.^11^

In accordance with current BrS drug risk stratification, drugs were grouped as being “drugs to avoid” and “drugs to preferably avoid.”^11^ “Drugs to avoid” have been associated with arrhythmias and the typical (type 1) BrS ECG. It is strongly advised to avoid these drugs in patients with BrS or to use these drugs only after extensive consideration and/or in controlled conditions. Most of the drugs in this list are associated with a Class IIa recommendation to avoid these drugs. “Drugs to preferably avoid” have been associated with the typical (type 1) BrS ECG pattern, but there is not yet substantial evidence that these drugs can cause malignant arrhythmias. In the “drugs to preferably avoid” category are also drugs for which only experimental evidence suggests a possible deleterious effect in BrS, and it is generally a Class IIb recommendation to avoid these drugs. However, it is still advised to consider avoiding these drugs or to use them only after extensive consideration and/or in controlled conditions.

### ICD Data

All Danish patients with an ICD implanted are registered in the Danish Pacemaker and ICD Register. This register holds nationwide information on implant and explant and information on follow‐up including ICD therapy (ie, appropriate and inappropriate shocks and anti‐tachycardia pacing). We used the date of first appropriate therapy (either anti‐tachycardia pacing, shock therapy, or both) after the patient's diagnosis of BrS.^12^ Delayed implantation of an ICD (ie, implantation of an ICD more than 3 months after a patient's BrS diagnosis date) was defined as a marker for an arrhythmic event.

### Other Covariates

Information on patient comorbidity up to 5 years before time of BrS diagnosis was obtained through the Danish National Patient Registry. Here we gathered information on relevant comorbidities according to the Charlson comorbidity index.^13^ Information on any psychiatric disease was also identified. Concomitant pharmacotherapy in the 90 days leading up to diagnosis was identified through the Register of Medicinal Products Statistics of the Danish Medicines Agency. Diabetes was defined as either presence of a diagnosis code for diabetes in the registries before diagnosis or a dispensed prescription of an antidiabetic drug within the 180 days leading up to diagnosis.^14^ Hypertension was defined as having dispensed prescriptions of 2 or more antihypertensive drugs within the 180 days leading up to diagnosis, as done previously.^15^ Information on the specific *ICD‐10* codes and Anatomical Therapeutic Chemical codes used to define patient comorbidity and concomitant pharmacotherapy are listed in Table S1.

### Statistical Analysis

Continuous variables were compared using the Kruskall‐Wallis test and categorical variables using the *χ* ^2^ test or Fisher' exact test where appropriate. Factors associated with treatment with any nonrecommended BrS drugs after diagnosis of BrS were identified using multivariable Cox proportional hazards regression model with time from diagnosis as the underlying time scale. End of study was defined by first claimed prescription of a nonrecommended BrS drug, passing the end of the observational period (December 31, 2019), loss to follow‐up (eg, emigration), or death, whichever came first. Variables included in the model were age at diagnosis (5‐year increment), sex, year of diagnosis, disease manifestation (ie, symptomatic or asymptomatic or unspecified), any psychiatric disease at baseline, any nonrecommended BrS drug use in the 90 days before diagnosis with BrS, and patient comorbidity burden (ie, Charlson comorbidity index) at time of diagnosis. Associations between any nonrecommended BrS drug use and appropriate ICD therapy, mortality, or a combined end point of an adverse event (ie, delayed ICD implantation, appropriate ICD therapy, and mortality), were assessed using multivariate logistic regression models including the same variables as mentioned above.

Statistical analyses were performed using SAS, version 9.4 (SAS Institute Inc, Cary, NC) and R Core Team (2022), version 4.0.3. For all analyses, a 2‐sided *P* value <0.05 was considered statistically significant.

### Ethics

The present study was approved by the Danish Data Protection Agency (P‐2019‐348). Registry‐based analyses using de‐identifiable data are exempt from ethics approval in Denmark. Collection of additional clinical data for a subset of the patients was approved by the regional ethics committee (journal‐nr.: H‐17032105) and with consent from the patients. Approval of the use of data from the Danish Pacemaker and ICD Register was obtained (DPICD‐2022‐06‐25).

## RESULTS

### Patient Characteristics

We identified 270 patients with BrS diagnosed between July 1, 2006 and December 31, 2018 and eligible for a minimum of 12 months of follow‐up through the registries. The median age at diagnosis was 46.1 years (interquartile range [IQR] 32.6–57.4), and 70.4% of patients were male. Patients were followed for a median of 6.8 years (IQR 3.7–9.0). Baseline characteristics are listed in Table 1. More than one‐third of patients (n=97, 35.9%) had an ICD implanted either before or after BrS diagnosis. A total of 6.3% (17/270) of patients were treated with any nonrecommended drug in the 90 days before the time of diagnosis with BrS. Overall, we found the disease manifestation before diagnosis with BrS to be aborted cardiac arrest in 31 patients (11.5%), and ventricular tachycardia or syncope in 92 patients (34.1%). There were 19 (7.0%) patients known to be asymptomatic, and 128 (47.4%) patients had an unspecified disease manifestation (Table 1). There were no significant differences in baseline characteristics of patients with and without an ICD besides differences in disease manifestation (Table S2).

**Table 1:** Baseline Characteristics of Patients With Brugada Syndrome

	Patients with Brugada syndrome, n=270
Sex, male	190 (70.4%)
Age at diagnosis, y, median [IQR]	46 [33–57]
Disease manifestation	
Asymptomatic	19 (7.0%)
Unspecified	128 (47.4%)
Syncope or ventricular tachycardia	92 (34.1%)
Aborted cardiac arrest	31 (11.5%)
ICD implanted	97 (35.9%)
Comorbidities before diagnosis	
Charlson comorbidity index ≥1	16 (5.9%)
Diabetes	8 (3.0%)
Hypertension	26 (9.6%)
Any psychiatric disease	16 (5.9%)
Ischemic heart disease	18 (6.7%)
Atrial fibrillation	17 (6.3%)
Epilepsy	5 (1.9%)
Cancer	8 (3.0%)
Concomitant pharmacotherapy*	
β‐Blockers	26 (9.6%)
Diuretics	24 (8.9%)
Antidepressants	17 (6.3%)
Antipsychotics	8 (3.0%)
Nonrecommended BrS drugs	
A drug to avoid	6 (2.2%)
A drug to preferably avoid	12 (4.4%)
Any of the 2	17 (6.3%)

BrS indicates Brugada syndrome; ICD, implantable cardioverter defibrillator; and IQR, interquartile range.

* 90 days before diagnosis.

For a subset of patients (n=67), additional clinical information from chart review was available (Table S3). Among patients with BrS eligible for chart review, 48 patients (71.6%) were probands, 58 patients (86.6%) had had a genetic test performed, and in 14 of these (24.1%) a pathogenic mutation or a likely pathogenic mutation was identified, all of which were *SCN5A* mutations. A total of 37 patients (55.2%) had a spontaneous type 1 BrS ECG. There were no significant differences in baseline characteristics between all patients with BrS and patients with BrS with chart review available (Table S3).

### Nonrecommended Drugs During Follow‐Up

A total of 34.4% (93/270) patients with BrS were treated with any nonrecommended BrS drug during a median follow‐up of 6.8 years after they had been diagnosed with BrS. The most commonly first prescribed nonrecommended BrS drugs were tramadol (n=44), fexofenadine (n=18), metoclopramide (n=14), and propranolol (n=5), which are all drugs to preferably avoid. Other prescribed drugs to preferably avoid were verapamil, carbamazepine, lamotrigine, fluoxetine, paroxetine, and bupropion, all prescribed to ≤3 patients (Table 2). Four patients were treated with a drug to avoid as the first prescribed drug (lithium or nortriptyline), and a total of 8 patients were treated with 1 or more drugs to avoid (lithium, nortriptyline, amitriptyline, propafenone, flecainide, or cannabis) during follow‐up. A total of 90 patients were treated with drugs to preferably avoid. Twenty‐three patients (24.7%) were treated with ≥2 different nonrecommended BrS drugs during follow‐up. Of the 17 patients treated with nonrecommended drugs in the 90 days immediately before BrS diagnosis, 13 (76.5%) continued use of the same drug (n=8) or were treated with other types of nonrecommended drugs (n=5) after their BrS diagnosis.

**Table 2:** List of Prescribed Nonrecommended Drugs After a Diagnosis of Brugada Syndrome

Drug	Number of patients treated as their first prescribed drug	Total number of patients treated during follow‐up	Type
Tramadol	44	52	Preferably avoid
Fexofenadine	18	21	Preferably avoid
Metoclopramide	14	22	Preferably avoid
Propranolol	5	5	Preferably avoid
Lamotrigine	≤3	6	Preferably avoid
Lithium	≤3	≤3	Avoid
Nortriptyline	≤3	≤3	Avoid
Bupropion	≤3	≤3	Preferably avoid
Carbamazepine	≤3	≤3	Preferably avoid
Fluoxetine	≤3	≤3	Preferably avoid
Paroxetine	≤3	≤3	Preferably avoid
Verapamil	≤3	≤3	Preferably avoid
Amitriptyline	…	4	Avoid
Cannabis	…	≤3	Avoid
Flecainide	…	≤3	Avoid
Propafenone	…	≤3	Avoid
Imipramine	…	≤3	Preferably avoid

**Figure 1:**
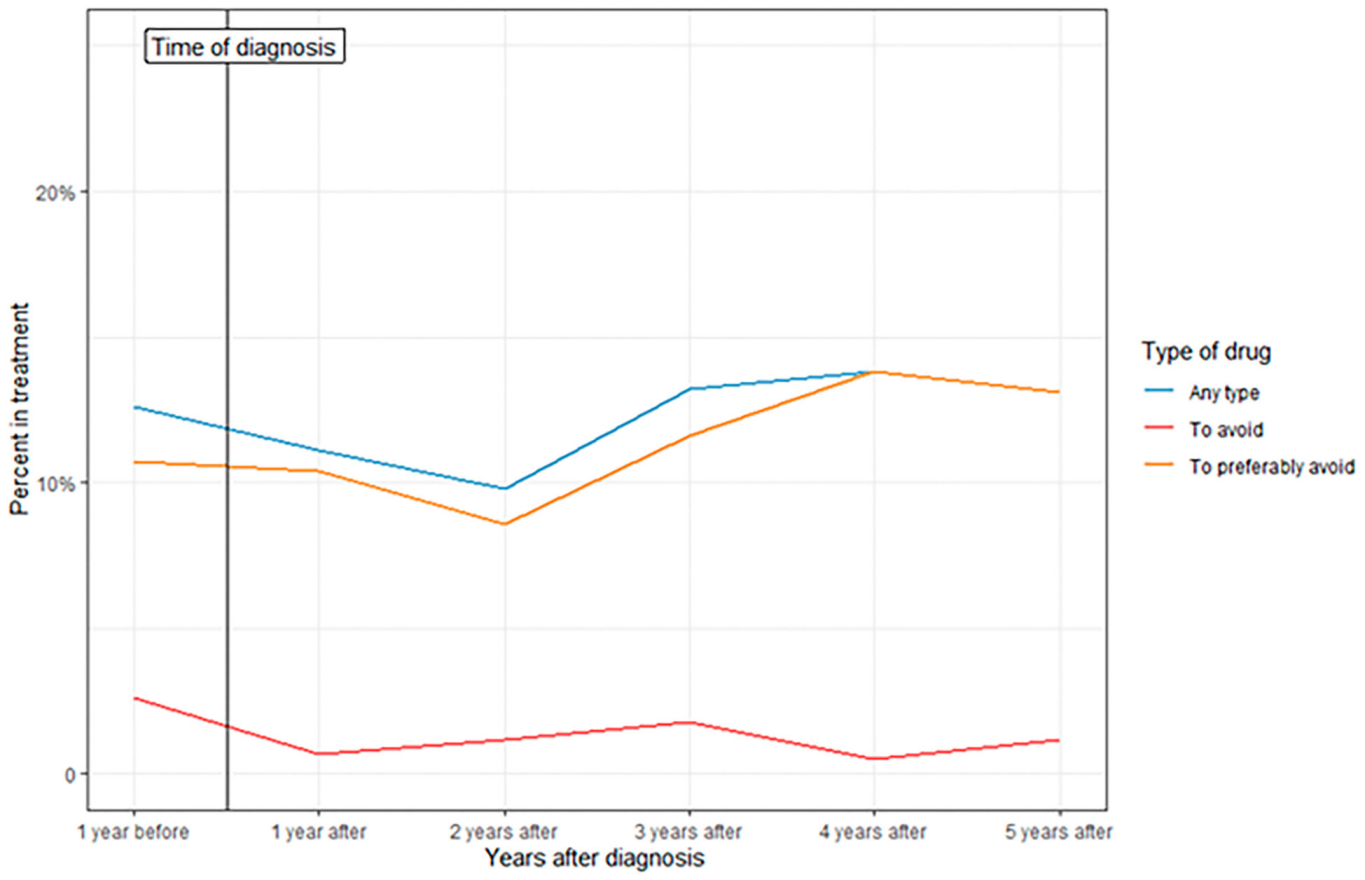
Proportion of patients with BrS in treatment with nonrecommended drugs before and after time of BrS diagnosis. BrS indicates Brugada syndrome.

In the year before diagnosis, 34 patients (12.6%) were treated with 1 or more nonrecommended BrS drugs (2.6% were treated with a drug to avoid, and 10.7% were treated with a drug to preferably avoid). Included in these numbers are the ≤3 patients who were treated with both a drug to avoid and a drug to preferably avoid. No difference in the proportion of patients receiving any nonrecommended BrS drug before the time of diagnosis with BrS (12.6%) compared with each of the 5 years following BrS diagnosis was identified (1 year: 11.1%; 2 years: 9.8%; 3 years: 13.2%; 4 years: 13.8%; 5 years: 13.1%; *P*>0.05 for all compared with 1 year before diagnosis; Figure 1).

Patients treated with nonrecommended BrS drugs during follow‐up were more likely to have a greater comorbidity burden (ie, Charlson comorbidity index ≥1 [10.8% versus 3.4%, *P*=0.03] and psychiatric disease [11.8% versus 2.8%, *P*=0.007]; Table 3).

**Table 3:** Baseline Characteristics of Patients With BrS Stratified by Nonrecommended Drug Use During Follow‐Up

	No nonrecommended drug use during follow‐up, n=177	Nonrecommended drug use during follow‐up, n=93	*P* value
Sex, male	132 (74.6%)	58 (62.4%)	0.05
Age at diagnosis, y, median [IQR]	45.1 [32.6–57.0]	47.8 [31.7–58.4]	0.4
Disease manifestation			0.2
Asymptomatic	16 (9.0%)	≤3	
Unspecified	82 (46.3%)	46 (49.5%)	
Syncope or ventricular tachycardia	56 (31.6%)	36 (38.7%)	
Aborted cardiac arrest	23 (13.0%)	8 (8.6%)	
ICD implanted	60 (33.9%)	37 (39.8%)	0.4
Comorbidities before diagnosis			
Charlson comorbidity index ≥1	6 (3.4%)	10 (10.8%)	0.03
Diabetes	4 (2.3%)	4 (4.3%)	0.6
Hypertension	19 (10.7%)	7 (7.5%)	0.5
Any psychiatric disease	5 (2.8%)	11 (11.8%)	0.007
Ischemic heart disease	10 (5.6%)	8 (8.6%)	0.5
Atrial fibrillation	11 (6.2%)	6 (6.5%)	1
Epilepsy	≤3	4 (4.3%)	0.09
Cancer	4 (2.3%)	4 (4.3%)	0.6
Concomitant pharmacotherapy*			
β‐Blockers	17 (9.6%)	9 (9.7%)	1
Diuretics	15 (8.5%)	9 (9.7%)	0.9
Antidepressants	6 (3.4%)	11 (11.8%)	0.01
Antipsychotics	4 (2.3%)	4 (4.3%)	0.6
Nonrecommended BrS drugs			
A drug to avoid	≤3	≤3	0.7
A drug to preferably avoid	≤3	10 (10.8%)	<0.001
Any of the 2	≤3	12 (12.9%)	<0.001

BrS indicates Brugada syndrome; ICD, implantable cardioverter defibrillator; and IQR, interquartile range.

* 90 days before diagnosis.

### Factors Associated With Treatment With Nonrecommended Drugs During Follow‐Up

**Figure 2:**
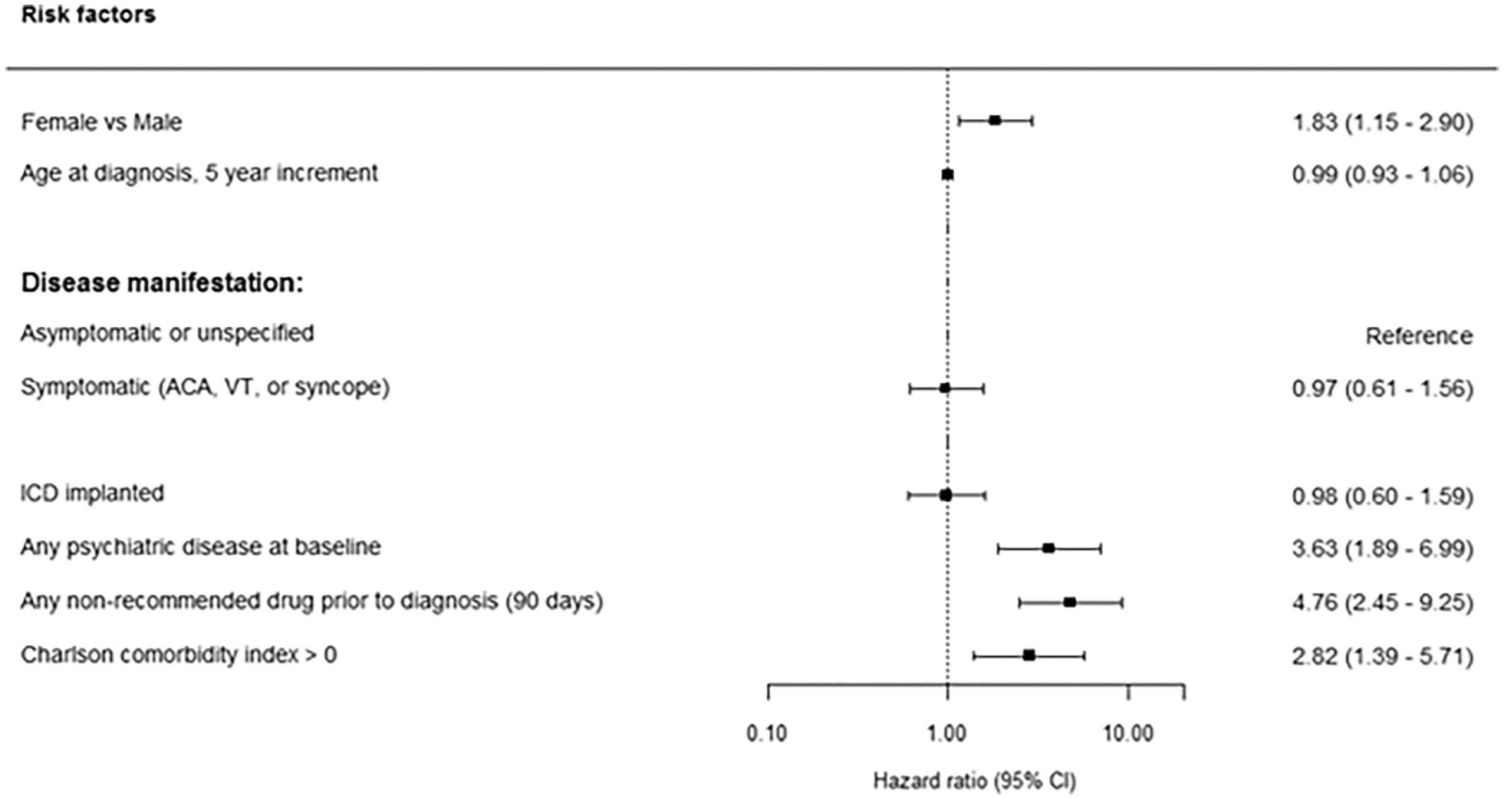
Factors associated with any nonrecommended BrS drug use after diagnosis. Cox proportional hazards model additionally adjusted for year of diagnosis. ACA indicates aborted cardiac arrest; BrS, Brugada syndrome; ICD, implantable cardioverter defibrillator; and VT, ventricular tachycardia.

Results from the Cox proportional hazards regression model are depicted in Figure 2. We found that female sex was associated with an increased risk of treatment with any nonrecommended BrS drug after the time of BrS diagnosis compared with male sex (hazard ratio [HR], 1.83 [95% CI, 1.15–2.90]). Furthermore, psychiatric disease (HR, 3.63 [95% CI, 1.89–6.99]), any nonrecommended BrS drug use within 90 days before diagnosis (HR, 4.76 [95% CI, 2.45–9.25]), and patients with a more significant comorbidity burden (ie, Charlson comorbidity index ≥1, HR, 2.82 [95% CI 1.39–5.71]) were also associated with increased risk of treatment with any nonrecommended BrS drugs after diagnosis.

### ICD Therapy During Follow‐Up

A total of 97 patients received an ICD; 30 patients received an ICD before date of BrS diagnosis, and 67 after time of BrS diagnosis. Patients who received their ICD before BrS diagnosis had a median time from implantation to diagnosis of 1305 days [IQR 61–2498], whereas patients who received their ICD after BrS diagnosis had a median time from diagnosis to implantation of 8 days [IQR 5–43]. Twenty patients experienced appropriate therapy (ie, shock or anti‐tachycardia pacing) after they had been diagnosed with BrS. Twelve patients received 1 or more shock therapies. Five patients received inappropriate therapy. Eight of the 20 patients who received appropriate therapy were treated with a nonrecommended BrS drug during follow‐up, but none of them within 30 days of appropriate ICD therapy (ie, they did not claim a prescription for a nonrecommended BrS drug <30 days of appropriate therapy). Among patients with an ICD implanted, no significant association between any nonrecommended BrS drug use at any time and appropriate ICD therapy was identified, although numbers were small (odds ratio [OR], 0.7 [95% CI, 0.2–2.4], *P*=0.6).

### Mortality and Combined Arrhythmic End Point During Follow‐Up

During follow‐up, 10 patients (3.7%) died (8 were male, and the median age at death was 68.7 years [IQR, 63.2–76.6 years]). None of the ≤3 patients who had an ICD implanted and died, received device therapy. Seven of the patients who died were treated with 1 or more nonrecommended BrS drugs during follow‐up, and 4 within 30 days of their death. Few patients (≤3) received 2 different nonrecommended BrS drugs, and the rest received 1 nonrecommended BrS drug. Three or fewer patients received drugs to avoid (nortriptyline or amitriptyline), and the rest received drugs to preferably avoid (tramadol, verapamil, or metoclopramide). No significant association between any nonrecommended BrS drug use after diagnosis and risk of all‐cause mortality was identified (OR, 3.39 [95% CI, 0.7–19.6], *P*=0.14); however, events were few.

A total of 38 patients either had delayed implantation of an ICD, received appropriate ICD therapy, or died (during follow‐up). There was no significant association between any nonrecommended BrS drug use during follow‐up and the combined end point (OR, 1.7 [IQR, 0.8–3.7], *P*=0.19).

### Sensitivity Analysis

To test the robustness of our results, we repeated the Cox proportional hazards regression model excluding the 17 patients who were treated with any nonrecommended BrS drug in the 90 days before diagnosis. The analysis yielded similar results as the main analysis, including female sex (HR, 2.08 [95% CI, 1.28–3.38]) and any psychiatric disease at baseline (HR, 2.65 [95% CI, 1.23–5.79]) that both remained significantly associated with any nonrecommended BrS drug use during follow‐up (Figure S1).

## DISCUSSION

The present nationwide study on pattern of prescriptions of nonrecommended BrS drugs among patients with BrS after the time of diagnosis had 3 principal findings. First, we found that treatment with any nonrecommended BrS drugs after the time of diagnosis was common among patients with BrS. More than one‐third of patients were treated with at least 1 drug during follow‐up, of which the majority were drugs to preferably avoid and to a lesser extent drugs to avoid. We did not identify any difference in the proportion of patients being prescribed nonrecommended BrS drugs on a yearly basis before and 5 years after the time of diagnosis. Second, female sex, any nonrecommended BrS drug use before diagnosis, and any psychiatric disease at diagnosis were associated with any nonrecommended BrS drug use after a diagnosis of BrS. Third, 7 out of 10 patients who died during follow‐up were treated with any nonrecommended BrS drugs during follow‐up; however, no significant association between any nonrecommended BrS drug use after diagnosis and mortality was identified, but events were few.

Surprisingly, one‐third of patients diagnosed with BrS were treated with a nonrecommended BrS drug during follow‐up in the present study; 8 patients were treated with 1 or more drugs to avoid, and 90 patients were treated with 1 or more drugs to preferably avoid. Moreover, there was no change in prescription patterns after time of BrS diagnosis, with approximately one‐third of all patients with BrS being treated with any nonrecommended BrS drug up to 5 years after time of diagnosis (Figure 1). This is of concern since it is a Class I recommendation that patients with BrS should not be treated with these drugs.^3^ Most of the drugs the patients in our study were treated with are listed in the “preferably avoid” group (tramadol, metoclopramide, and fexofenadine), which generally have conflicting evidence as to their arrhythmogenicity (Class IIb recommendation). In 2019, a similar study on the intake of drugs not recommended for use in patients with congenital long QT syndrome was published.^8^ Here, almost 60% of the patients with congenital long QT syndrome had used a drug that was not recommended for use in patients with congenital long QT syndrome because of either QT‐prolonging properties or the risk of inducing torsades de pointes ventricular tachycardia. In both cases, prescribing physicians may not be aware of the recommendations on drugs to avoid in the respective diseases, even though patients most likely have been informed of this. In Denmark, no direct communication between the cardiologist and all physicians treating a certain patient or clinical decision support tools to detect drug‐diagnosis interactions exist, and thus it is important that the patients be involved in their treatment. In some cases, the prescribing physician may be aware of the recommendations and have made a weighted choice of prescribing a certain drug despite its possible consequences in relation to the cardiac disease, because it may be the best or only treatment for another disease for the patient. This may be part of the explanation for the association we found between psychiatric disease at baseline and any nonrecommended BrS drug use. Additionally, we found that previous nonrecommended BrS drug use was significantly associated with continued use of nonrecommended BrS drugs after the diagnosis of BrS, and that 76.5% of patients treated with nonrecommended drugs before diagnosis continued use of the same or another nonrecommended drug after diagnosis. Any previous or current nonrecommended BrS drug use should therefore be considered by the cardiologist diagnosing the patient, to discontinue further use of these drugs at the time of BrS diagnosis, if possible.

In the present study, we did not identify a significant association between use of nonrecommended BrS drug after diagnosis and appropriate ICD therapy; however, the number of events was low, and the patients were primarily treated with drugs to preferably avoid, which generally have weak evidence for arrhythmogenicity in patients with BrS. A Portuguese study from 2018 on 30 patients with BrS and an ICD implanted found that 53.3% of the included patients had taken a nonrecommended drug, and that 6 out of 7 patients (85.7%) who had experienced appropriate ICD therapies had received a nonrecommended drug as opposed to 45.4% of patients without ICD therapies (difference borderline significant, *P*=0.062).^4^ The authors found a mean time between therapies and unsafe drug use of 3.8±7.5 days. In our study, we found none of the registered appropriate ICD therapies to be preceded by a new prescription of any nonrecommended BrS drug, even though there were no significant differences in the types of nonrecommended drugs patients were treated with in the 2 studies. However, 7 out of 10 patients with BrS, who died during follow‐up, were treated with a nonrecommended BrS drug after their diagnosis at some point, and 4 of them had redeemed a prescription within 30 days of their time of death. We found an OR of 3.39 for association between mortality and nonrecommended drug use; however, this was not significant (*P*=0.14). In comparison, a study from 2017 analyzed drug use before sudden cardiac death and found a 2‐fold increase in the risk of sudden arrhythmic death syndrome in individuals treated with a nonrecommended BrS drug.^16^

### Limitations

The present study is a retrospective register‐based study and has limitations inherent to this. Despite efforts that were made to adjust for confounders, we cannot exclude the risk of confounding by indication or that residual confounding may have influenced our findings. Moreover, despite the fact that our study is a nationwide study including all Danish patients diagnosed with BrS from 2006 to 2018, the sample size is small because BrS is a rare disease.^17^ The diagnosis code for BrS was implemented in the Danish registries in 2006, which means that there is a potential latency between a diagnosis of BrS before 2006 and registration in the nationwide registries, and this may have influenced some of our findings. For patients with charts available for review, we were able to assess the date of diagnosis through medical records. This resulted in adjusted diagnosis dates for 5 patients who were diagnosed before 2006, the earliest being adjusted to 2002. In general, the nationwide registries do not contain clinical information on patients, and thus, we only had access to information on proband status, genotype, and whether patients were diagnosed by a spontaneous type 1 BrS ECG in patients with charts available for review.

The brugadadrugs.org website was created in May 2009, and the first official publication was published in September 2009.^11^ However, it is a dynamic list, and drugs have since been added to the list. For example, in 2011 tramadol was added to the preferably avoid list and oxcarbazepine was added to the avoid list. Thus, if a patient diagnosed with BrS was treated with tramadol in 2010, it was not against the recommendations at that time point. The latest additions were made in January 2015. In this study we have examined the use of the drugs that were on the list from the most recent update in all patients, and we cannot exclude that this may have influenced our results. Additionally, many antihistamines, including some drugs containing fexofenadine, can be bought without a prescription at Danish pharmacies (ie, over the counter) and would not be registered. The number of patients in our study having taken this drug at some point is therefore potentially lower than the real number. Patients with a more sporadic need for antihistamines may not get a prescription; however, patients with chronic allergic illness are eligible for partial reimbursement and thus have an economic incentive to have the drug prescribed. In general, we assume that patients who claimed a prescription for a drug were also likely to take it because of them having an economic incentive to do so.

We found no statistically significant association between any nonrecommended BrS drug use and all‐cause mortality; however, our analysis was limited by a low number of outcomes. It is important to note that even though prescription of a nonrecommended BrS drug preceded all‐cause mortality, the current study is observational, and thus we cannot conclude causality between the two.

## CONCLUSIONS

In our nationwide study of 270 Danish patients with BrS, more than one‐third of patients with BrS were treated with at least 1 nonrecommended BrS drug after the time of diagnosis; however, only a few of these patients were treated with drugs in the “avoid” group. No change in overall use of any nonrecommended BrS drug was identified between the year before diagnosis and the 5 years following diagnosis of BrS. Female sex, psychiatric disease at baseline, and previous use of nonrecommended BrS drugs were significantly associated with any nonrecommended BrS drug use after diagnosis. No significant association between any nonrecommended BrS drug use after diagnosis and mortality was identified; however, the number of patients who died was small. Although most patients were treated with drugs to preferably avoid, our findings warrant more awareness of prescription patterns in patients with BrS, in whom treatment with nonrecommended BrS drugs may increase the risk of ventricular arrhythmias and sudden cardiac death.

## Sources of Funding

The project was supported by the Novo Nordisk foundation (Tandem Programme; #31634) and the John and Birthe Meyer foundation.

## Disclosures

Prof. Torp‐Pedersen reports a grant from Bayer for randomized trials and from Novo Nordisk for epidemiological study, all unrelated to the present work. Prof. Køber reports honoraria from Novo Nordisk, Novartis, AstraZeneca, and Boehringer, unrelated to this article. All remaining authors have declared no conflicts of interest.

## Supporting information

Tables S1–S3
Figure S1
