# Correction to: Use of Nonrecommended Drugs in Patients With Brugada Syndrome: A Danish Nationwide Cohort Study

**DOI:** 10.1161/JAHA.122.027688

**Published:** 2023-05-15

In the article by Camilla H. B. Jespersen et al, “Use of Nonrecommended Drugs in Patients With Brugada Syndrome: A Danish Nationwide Cohort Study,” which published online March 21, 2023 (*J Am Heart Assoc*. 2023;12:e028424. DOI: 10.1161/JAHA.122.028424) and was included in the April 4, 2023 issue of the journal, a correction was needed.

The name of the 16th author, Jacob Tfelt‐Hansen, was incorrectly written in the published article as Jacob Tfelt Hansen. This error has now been corrected.

The authors regret the error.

The online version of the article has been updated and is available here: https://www.ahajournals.org/doi/10.1161/JAHA.122.028424.

