# Supplementary material for: Use of Nonrecommended Drugs in Patients With Brugada Syndrome: A Danish Nationwide Cohort Study
Source: J Am Heart Assoc. 2023 Mar 21;12(7):e028424. doi: 10.1161/JAHA.122.028424 (PMC10122907; doi:10.1161/JAHA.122.028424)
Supplement: Supplementary file 1 — Tables S1–S3 Figure S1 [file JAH3-12-e028424-s001.pdf]

SUPPLEMENTAL MATERIAL

Table S1. Codes for treatment and definition of diagnoses

Diagnoses/comorbidities	ICD-10 codes
Aborted cardiac arrest	I460, I469, I490, I490B
Any psychiatric disease	F
Atrial fibrillation	I48
Epilepsy	G40, G41
Ischemic heart disease or prior myocardial infarction	I20-25
Syncope	R559
Ventricular tachycardia	I470, I472, I472A, I472B, I472D

Therapy	ATC codes
ACE-inhibitors	C09A
Antiarrhythmic drugs	C01B
Antidepressants	N06A
Anxiolytics	N05B, N05C
Beta blockers	C07
Calcium channel blockers	C08
Lipid lowering drugs	C10
Loop diuretics	C03C
Thiazides	C03A

Comorbidities based (partly) on ATC codes	ATC or ICD-10 codes
Diabetes[14]	Diagnosis code within 5 years of diagnosis of ICD10: E10-E14 *and/or*<br>A dispensed prescription within 180 days of diagnosis of the following:<br>ATC: A10

Hypertension[15]	2 or more dispensed prescriptions of the following within 180 days of diagnosis (ATC):<br>α adrenergic blockers: C02A, C02B, C02C<br>Non-loop diuretics: C02DA, C02L, C03A, C03B, C03D, C03E, C03X, C07B, C07C, C07D, C08G, C09BA, C09DA, C09XA52<br>Vasodilators: C02DB, C02DD, C02DG<br>β blockers: C07<br>Calcium channel blockers: C07F, C08, C09BB, C09DB<br>Renin–angiotensin system inhibitors: C09

Table S2. Baseline characteristics of patients with BrS stratified by ICD

	Patients with BrS and an ICD, n=97	Patients with BrS and no ICD, n=173	p-value
**Sex (male)**	74 (76.3%)	116 (67.1%)	0.1
**Age at diagnosis, years (median [IQR])**	46 [34.6-57.6]	46.9 [29.2-57.3]	0.5
**Disease manifestation**			<0.001
Asymptomatic	0	19 (11.0%)	
Unspecified	24 (24.7%)	104 (60.1%)	
Syncope or ventricular tachycardia	44 (45.4%)	48 (27.7%)	
Aborted cardiac arrest	29 (29.9%)	≤3	
**Comorbidities prior to diagnosis**			
Charlson comorbidty index ≥1	8 (8.2%)	8 (4.6%)	0.3
Diabetes	4 (4.1%)	4 (2.3%)	0.6
Hypertension	8 (8.2%)	18 (10.4%)	0.7
Any psychiatric disease	6 (6.2%)	10 (5.8%)	1
Ischemic heart disease	9 (9.3%)	9 (5.2%)	0.3
Atrial fibrillation	7 (7.2%)	10 (5.8%)	0.8
Epilepsy	4 (4.1%)	≤3	0.1
Cancer	≤3	6 (3.5%)	0.8
**Concomitant pharmacotherapy***			
Beta blockers	13 (13.4%)	13 (7.5%)	0.2
Diuretics	8 (8.2%)	16 (9.2%)	1
Antidepressants	7 (7.2%)	10 (5.8%)	0.8
Antipsychotics	≤3	5 (2.9%)	1
Non-recommended BrS drugs			
- A drug to avoid	≤3	≤3	0.8
- A drug to preferably avoid	≤3	9 (5.2%)	0.5
- Any of the two	6 (6.2%)	9 (5.2%)	1

*90 days prior to diagnosis

Abbreviations: BrS, Brugada Syndrome; ICD, implantable cardioverter defibrillator; IQR, interquartile range

Table S3. Baseline characteristics of patients with BrS stratified by whether charts were available for review

	Chart review not available, n=203	Chart review available, n=67	p-value
**Sex (male)**	143 (70.4%)	47 (70.1%)	1
**Age at diagnosis, years (median [IQR])**	45.3 [31.4-57.7]	47.2 [33.6-56.7]	0.7
**Disease manifestation**			<0.001
Asymptomatic	0	19 (28.4%)	
Unspecified	111 (54.7%)	17 (25.4%)	
Syncope or ventricular tachycardia	67 (33.0%)	25 (37.3%)	
Aborted cardiac arrest	25 (12.3%)	6 (9.0%)	
**Genetic test performed**	-	58 (86.6%)	
Mutation detected (*SCN5A*)	-	14 (24.1%)	
**Proband**	-	48 (71.6%)	
Spontaneous type 1 ECG	-	33 (68.8%)	
**Relative**	-	19 (28.4%)	
Spontaneous type 1 ECG	-	4 (21%)	
**ICD implanted**	75 (36.9%)	22 (32.8%)	0.6
**Comorbidities prior to diagnosis**			
Charlson comorbidity index ≥1	13 (3.4%)	≤3	0.8
Diabetes	6 (3.0%)	≤3	1
Hypertension	16 (7.9%)	10 (14.9%)	0.1
Any psychiatric disease	11 (5.4%)	5 (7.5%)	0.8
Ischemic heart disease	14 (6.9%)	4 (6.0%)	1
Atrial fibrillation	13 (6.4%)	4 (6.0%)	1
Epilepsy	≤3	≤3	0.8
Cancer	7 (3.4%)	≤3	0.7
**Concomitant pharmacotherapy***			
Beta blockers	19 (9.4%)	7 (10.4%)	1
Diuretics	16 (7.9%)	8 (11.9%)	0.4
Antidepressants	12 (5.9%)	5 (7.5%)	0.9
Antipsychotics	5 (2.5%)	≤3	0.7
Non-recommended BrS drugs			
- A drug to avoid	4 (2.0%)	≤3	1

	Chart review not available, n=203	Chart review available, n=67	p-value
- A drug to preferably avoid	8 (3.9%)	4 (6.0%)	0.5
- Any of the two	11 (5.4%)	4 (6.0%)	0.9

*90 days prior to diagnosis

Abbreviations: BrS, Brugada Syndrome; ICD, implantable cardioverter defibrillator; IQR, interquartile range

Figure S1. Risk factors associated with any non-recommended BrS drug use after diagnosis excluding patients treated with any non-recommended drug within 90 days of diagnosis with BrS

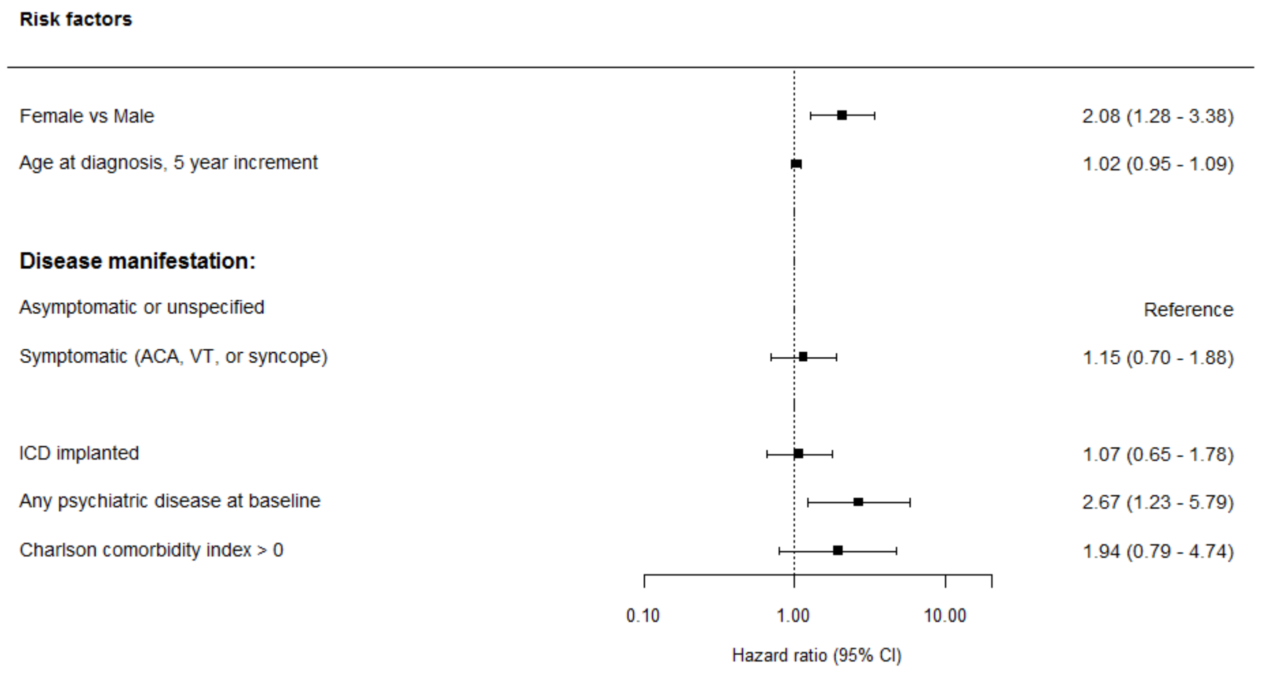

Cox proportional hazards model additionally adjusted for year of diagnosis.

Abbreviations: ACA, aborted cardiac arrest; CI, confidence interval; ICD, implantable cardioverter defibrillator; VT, ventricular tachycardia.
